# Mapping Small Ruminant Trade Networks in Ethiopia's Somali Region and Borena Zone: Implications for the Spread of Peste des Petits Ruminants Virus

**DOI:** 10.1155/tbed/6620243

**Published:** 2025-07-13

**Authors:** Asrat Arke Ashango, Hika Waktole, Samson Leta, Haileleul Negussie

**Affiliations:** ^1^Department of Veterinary Epidemiology and Public Health, College of Veterinary Medicine and Agriculture, Addis Ababa University, Bishoftu, Ethiopia; ^2^Department of Veterinary Microbiology, Parasitology and Poultry Health, College of Veterinary Medicine and Agriculture, Addis Ababa University, Bishoftu, Ethiopia; ^3^Department of Biomedical Sciences, College of Veterinary Medicine and Agriculture, Addis Ababa University, Bishoftu, Ethiopia

**Keywords:** livestock market, network analysis, PPRV, small ruminants

## Abstract

Livestock trade significantly contributes to the spread of transboundary animal diseases (TADs), like Peste des Petits Ruminants virus (PPRV), due to the long-distance movement of infected animals. This study investigates the structure and dynamics of the small ruminant trade network across 64 markets and 20 destinations, focusing on market connectivity, trade patterns, and disease risk. Eight key markets were identified due to their significant trading activity and connections. During the study period, 365,261 small ruminants were traded, with 54% directed toward market destinations and the remainder to slaughterhouses or cross-border locations, primarily Somaliland and Kenya. Over 85% of transport to these destinations was by truck, while more than 55% of internal movements were on foot. Trade peaked between July and September, accounting for 42% of the annual trade volume and 46% of cross-border movements. Network analysis revealed 172 distinct trade routes with varying activity levels: 12 persistent, 47 frequent, 42 intermediate, and 69 occasional. The network exhibited low connectivity (density = 0.024) and low reciprocity (0.104), with a negative assortativity (−0.52), indicating smaller markets often connected with larger ones. The network's clustering coefficient was 0.729, and the average path length was 2.216, contrasting with simulated random networks that had a clustering coefficient of 0.047 and an average path length of 3.22. Key markets were identified as “gate-keepers” and “pulse takers,” facilitating connections between isolated and central markets. The epidemic threshold (q) and R0 indicated a high risk of disease spread, especially from July to October. The study found that removing key markets drastically reduced network cohesion, with a 10% removal of central nodes decreasing the giant strongly connected component (GSCC) size by ~80%. Peste des Petits Ruminants (PPR) outbreaks in 2023 correlated strongly with market centrality, highlighting the need for targeted monitoring and control measures. This study underscores the importance of understanding network dynamics to manage trade efficiently and mitigate disease risks in small ruminant populations.

## 1. Introduction

Livestock movements, particularly through trade, are major contributors to the spread of infectious agents among animal populations. The movement of infected animals facilitates the long-distance transmission of pathogens, posing a significant challenge for controlling animal diseases [1, 2]. Effective disease control and surveillance require a deep understanding of livestock contact patterns, including the routes, volume, frequency, and risks associated with these movements [3]. The spread of animal diseases through trade involves various actors, such as farms, slaughterhouses, and traders, creating a complex network that is essential to understand for effective intervention [4]. Social network analysis (SNA) has emerged as a valuable tool to map these networks, identify key actors, and pinpoint high-risk areas, thereby improving our ability to control the spread of infectious diseases [5–9].

Ethiopia has a substantial small ruminant population, with an estimated 42.9 million sheep and 52.4 million goats [10]. Despite this, the country's contribution to foreign exchange earnings from livestock is lower than expected, largely due to the prevalence of transboundary animal diseases (TADs) such as Peste des Petits Ruminants (PPR), sheep and goat pox, and contagious caprine pleuropneumonia. PPR is a priority disease for small ruminants in Ethiopia, severely impacting food security and the livelihoods of livestock-dependent communities [11]. The failure to maintain control programs for PPR virus (PPRV), despite the availability of a highly effective vaccine since 1989, has led to the virus spreading to new regions and species, including camels [12]. Recognizing the severity of this threat, the World Organization for Animal Health (WOAH) and the Food and Agriculture Organization of the United Nations (FAO) launched the Global Control and Eradication Strategy (GCES) in 2013, aiming to eradicate PPR by 2030 [13].

To effectively control PPRV and other diseases, it is crucial to understand the determinants and dynamics of disease spread within complex trade networks [4]. Network-based approaches have been increasingly applied to study livestock movements in Africa, providing insights into the structure and vulnerabilities of these networks [3, 14–18]. Accurate analysis of trade-related movements, often derived from official movement permits, is essential for improving the identification and traceability of disease transmission routes [19].

In Ethiopia, PPRV is widespread and poses a significant challenge to small ruminant production [11]. Despite the high risk of transmission through trade, there is a lack of well-organized data on small ruminant trade structures, hindering effective surveillance and control efforts. This study aims to characterize the small ruminant trade network in the Borena Zone and Somali Region of Ethiopia, assess its potential role in the epidemiology of PPRV, and identify key markets and areas for targeted surveillance and control interventions. By analyzing the volume, frequency, and routes of small ruminant movements, this research seeks to enhance our understanding of the network's vulnerability to disease spread and inform more effective strategies for managing PPRV.

## 2. Materials and Methods

### 2.1. Study Area

The study was conducted in Borena Zone and Somali Region of Ethiopia. The study sites are selected purposively based on their livestock number, the presence of informal cross-border livestock trade, and their classification as high-risk or endemic for major transboundary diseases affecting small ruminants [11, 20, 21]. The estimated livestock population of Borena Zone is 420,512 sheep, 657,479 goats, 602,593 cattle, and 63,311 camels. The Somali region has an estimated livestock population of 6,034,887 cattle, 11,013,491 sheep, 16,464,505 goats, and 6,489,702 camels [10].

### 2.2. Study Approach

A network-based approach, adapted from SNA, was used to examine the structure and patterns of small ruminant movements through trade. This method helps understand network properties relevant to disease surveillance and control.

### 2.3. Data Collection Methods

Market Identification: Key livestock markets were identified through interviews with traders and livestock experts. Structured questionnaires were used, and interviews were conducted with knowledgeable individuals selected in consultation with regional trade and livestock offices.

Movement Data Collection: Data on animal trades, including source, destination, movement type, and volume, were recorded at markets. Additional information was gathered via phone calls and official records from trade offices. Retrospective Epidemiological Data: Data on PPR outbreaks for 2023 were obtained from the Ministry of Agriculture to assess the impact of market networks on PPRV incursion.

### 2.4. Data Analysis and Markets Network Construction

Data were entered into Excel for analysis. A fully weighted static network was constructed from January to December 2023, with markets as nodes and trade links as edges. Monthly networks were also created to capture seasonal variations. Network metrics and summary statistics were computed to assess network cohesiveness and centrality. Network topology was described using metrics like density, diameter, and clustering coefficient. Centrality measures, including in-degree, out-degree, betweenness, and eigenvector centrality, were calculated to identify key markets. Community detection was performed using the Infomap algorithm. The epidemic threshold (*q*) was calculated for monthly networks to assess the potential for disease spread. Percolation analysis was used to evaluate the impact of targeted node removal on network vulnerability. The Kruskal–Wallis test was used to analyze differences in trade volume among markets based on risk status. All analyses and visualizations were performed using R statistical software (igraph, ggplot2, mapMCDA packages) and QGIS (Version 3.30.2).

## 3. Results

### 3.1. Market Records Collection

Eight markets were selected for recording small ruminant trade movements due to their significant trading activity and extensive trade connections (Table 1). The livestock trade typically progresses from smaller, local markets near pastoral communities to larger, secondary markets in urban areas, to eventually reach final destinations such as slaughterhouses or cross-border markets.

### 3.2. Market Structure and Trade Movements

**Figure 1:**
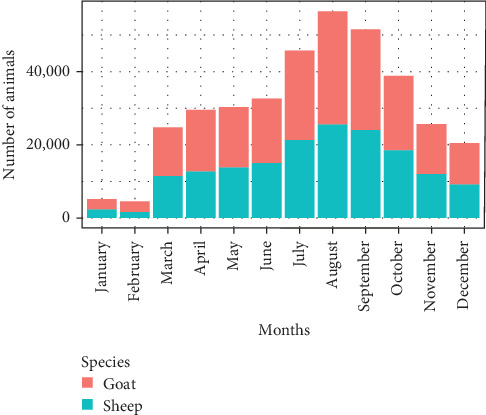
Volume of small ruminants traded through the network by month over 12 months.

**Figure 2:**
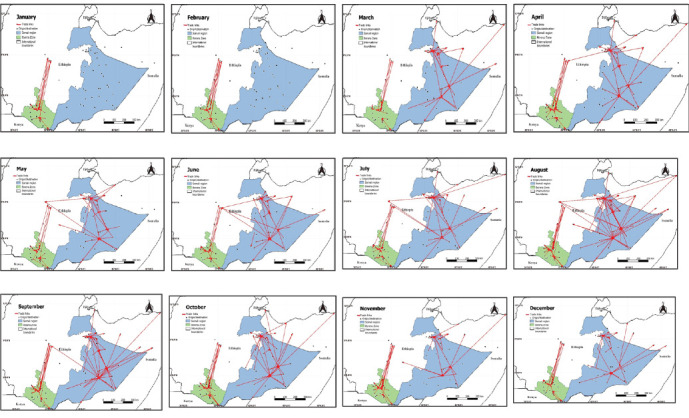
Monthly snapshot of small ruminant trade movement networks in the Borena Zone and Somali Region from January to December 2023. Each black dot represents the origin and destination points, and arrows represent trade links, with the heads of the arrows indicating destination points.

During the study period, 365,261 small ruminants were traded across 64 markets and 20 additional destinations. Over half (54%) of these movements were to market destinations, while the remaining were directed to final points like slaughterhouses and cross-border locations, primarily to Somaliland and Kenya (Table 2). Most animal transport (over 85%) to slaughterhouses and cross-border points was by truck, while more than 55% of internal movements were conducted on foot. The highest volume of trade exchange occurred from July to September, accounting for nearly 42% of the total annual trade, involving 153,119 animals (Figure 1). This period also saw the highest number of trade routes and accounted for 46% of cross-border movements (Figure 2).

### 3.3. Network Characteristics

The study identified 172 distinct trade routes with varying activity levels: 12 routes were active year-round (persistent), 47 were frequently active, 42 showed intermediate activity, and 69 were occasionally active (Figure S1). The trade network exhibited sparse connectivity, with a density of 0.024, indicating that only 2.4% of potential market pairs were connected. The reciprocity rate was low (0.104), suggesting a predominantly unidirectional flow from livestock keepers to final destinations. The degree of assortativity (connection preference) was –0.52, indicating a negative assortativity, where larger markets traded more frequently with smaller ones. The average path length between markets was 2.21 steps, with the longest path (diameter = 5) spanning markets in the Somali region to Kenya (Tables S1 and S2).

The degree distributions of markets in the small ruminant trade network were right-skewed (Figure S2). A small fraction of markets (Table S3) exhibited high connectivity; the top 10% accounted for 50% of connections, while the bottom 90% held the other 50%. Most markets (83%) had either only outgoing (out-degree) or incoming (in-degree) connections (Figure S3). The network's clustering coefficient and average path length were 0.729 and 2.216, respectively. In contrast, 1000 simulated random networks with the same number of nodes and links had an average path length of 3.22 and a clustering coefficient of 0.047.

### 3.4. Network Cohesiveness and Key Actor Analysis

**Figure 3:**
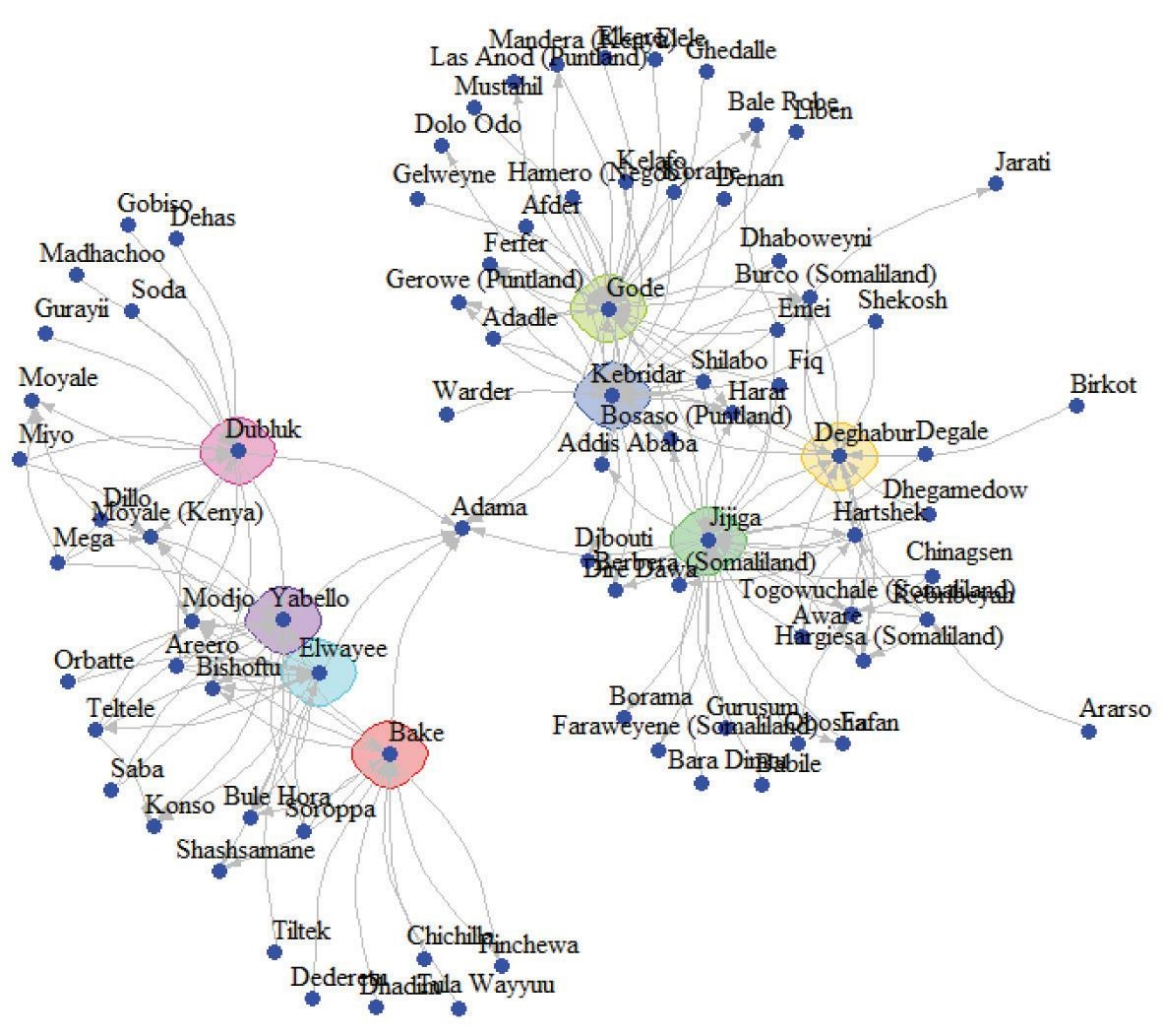
Core–periphery structure of small ruminant trade network in Borena (bottom left) and Somali region (top right). Markets colored with smooth polygons are core markets.

The small ruminant trade network centered around eight core markets, with 76 peripheral markets connected to them (Figure 3). All markets in the network were included in the giant weak component (GWCC). On the other hand, there were 76 strongly connected components (GSCC). The largest GSCC included 7 markets (Figure S5), while two strongly connected components contained two markets each, and the remaining 73 contained only one market. Community detection analysis revealed eight distinct market communities (Figure S4), with the largest community comprising 26 markets. Critical markets were identified based on the correlation coefficient among centrality measures (Table S4), with some acting as “gate-keepers” connecting isolated markets to the broader network, while others served as “pulse takers” facilitating access to central markets. Two markets, Deghabur and Yabello, were identified as “gate-keepers” because of their unique role in connecting more isolated markets to a broader trade network. Three markets, Elwaye, Kebribeyah, and Hartshek, were considered “pulse takers” due to their easy access to other central markets and the rest of the trade network, facilitated by their shortest paths. Five markets—Bake, Dubluk, Jijiga, Gode, and Kebridar—served dual roles as both “pulse takers” and “gate-keepers” (Figure S6).

### 3.5. Disease Spread Risk Assessment

The effect of changes in trade network structure on disease spread was assessed using the epidemic threshold (*q*) throughout the year. The entire small ruminant network was at risk year-round, with an average epidemic threshold (*q*) of 0.392 and an *R*_0_ of 2.75, indicating a highly contagious disease could spread to three markets from one (Figure S7b,c). The highest risk period was July to October (*q* < 0.4, *R*_0_ > 3), peaking in August when one infectious market could affect up to five others (Figure S7c). This period also saw the most trade activity, with 45% of movements and over 50% of occasional links (short-term routes) occurring, and 52.8% of animals traded (Figure S7a).

### 3.6. Network Vulnerability and Resilience

The impact of removing key nodes on network vulnerability and cohesion was assessed by sequentially deleting markets from the trade network based on their centrality scores. After each removal, network cohesion and vulnerability were measured using the size of the GSCC and the number of communities within the network. Removing markets with high in-degree, out-degree, and betweenness centrality scores led to a rapid decrease in network cohesion, with a 10% removal of the most central nodes reducing the GSCC size by about 80%. In contrast, random removal of over 50% of nodes was needed to achieve a similar effect (Figure S8).

### 3.7. PPR Outbreaks and Risk Status

In 2023, PPR outbreaks were reported in 22 districts within the study areas, with a strong correlation between PPR risk status and market centrality measures (Table S5 and Figure S1). Most markets (84%) were located in endemic or high-risk areas (Figure S10), which accounted for over 94% of annual trade volume (Table 3).

## 4. Discussion

Network-based approaches have become integral in veterinary epidemiology for analyzing how animal movements contribute to disease spread and for devising effective surveillance and control strategies [3, 22]. The importance of livestock trade in disease dissemination has garnered increased attention, as pathogens can be transmitted over long distances through the movement of infected animals [3]. Understanding contact patterns and the network's structure is crucial for identifying potential disease pathways and comprehending disease spread in complex systems. SNA techniques have been extensively used by veterinary epidemiologists to pinpoint central actors in the spread of infectious diseases [4]. This study investigated the small ruminant trade network and its susceptibility to the spread of PPRV in Ethiopia's major pastoral regions, Borena Zone and the Somali Region, using SNA combined with PPRV epidemiology.

The analysis revealed a clear seasonal pattern in the trade flow and volume of small ruminants, peaking between July and October 2023, when over 50% of the annual trade volume occurred. This peak is linked to seasonal factors influencing livestock market supply and demand. In Borena, the highest volume of cross-border trade is mainly attributed to prolonged drought until the spring of 2023 and increased demand for animals in the Middle East due to religious festivals [23]. Traders and pastoralists often sell their animals during this time to secure financial stability ahead of the upcoming dry season. In the Somali Region, higher demand during the autumn season was noted [24]. Additionally, seasonal factors like drought and festive periods, including the Hajj festival, influence both market availability and demand [25].

The small ruminant trade network exhibited low connectivity, with only 2.4% of potential links realized. This sparse network suggests that animal trade in Borena and Somali regions is mostly local, with limited destinations. The network's small diameter (5) and average path length (2.2) imply that markets are closely connected, potentially facilitating rapid disease spread if introduced [18]. The presence of hubs and low epidemic thresholds (average *q* = 0.39) further indicates the possibility of rapid disease dissemination [16, 26, 27].

The degree distribution of markets was highly skewed, with a small fraction of highly connected markets handling a significant portion of the trade. This heterogeneity reflects the central role of large urban markets as destinations for animals from smaller markets, including export destinations and cross-border trade. Markets with high out-degrees and betweenness centrality act as super-spreaders, while those with high in-degrees are at higher risk of infection [28, 29]. Targeting these key markets for surveillance, vaccination, and biosecurity measures could effectively reduce disease spread [30–32].

The observed network characteristics, high global clustering coefficient (0.73), and short path length (2.21) align with small-world network topology, known for rapid disease spread due to its clustered nature and few long-distance connections [3, 33, 34]. Previous studies have highlighted the susceptibility of small-world networks to swift disease dissemination [31, 35, 36].

The network's negative assortative mixing indicates that larger, more connected markets trade more frequently with smaller, less connected ones. This disassortative mixing can facilitate disease spread but also makes the network more amenable to targeted interventions, as disrupting highly connected nodes is more effective in reducing overall network cohesion [37, 38]. Thus, targeted control measures like vaccination and movement restrictions could be more resource-efficient than random interventions [4, 18, 19].

Analysis of epidemic threshold parameters revealed an average epidemic threshold (*q* = 0.392) and a Basic Reproduction Number (*R*_0_ = 2.75), indicating a high risk of disease spread. The lowest risk period was December (*q* = 0.6, *R*_0_ = 1.66), while the highest risk occurred from July to October, peaking in August (*q* = 0.199, *R*_0_ = 5), correlating with increased trade and festive periods [23–25]. Previous studies have also identified high risk during periods of increased trade volume and new market connections [16, 26, 39].

The Ministry of Agriculture's 2023 update identified 19 districts as endemic, 32 as high risk, and 12 as relatively risk-free. A strong correlation between PPR risk status and centrality measures (out-degree, out-weight, and eigenvector centrality) was observed, suggesting that markets with higher outgoing trade connections are in higher-risk areas. These high-risk areas are significant sources of animals traded through the network, increasing the risk of PPRV transmission [40, 41]. This highlights the urgent need to monitor and control trade routes as part of the current risk-based PPR control program, mitigating the spread of the disease in small ruminant populations. Targeted removal of markets based on centrality measures proved more effective than random removal in reducing network cohesiveness and epidemic potential. Removing the top 10% of markets based on centrality reduced the size of the GSCC by ~80%, whereas random removal required more extensive action. This finding supports previous research advocating for targeted node removal to control disease spread [3, 17, 28, 37].

In conclusion, this study provides a comprehensive assessment of small ruminant movement patterns in the Borena zone and the Somali regions, highlighting the role of highly connected hub markets and the network's small-world topology. Targeted interventions, focusing on key markets, could enhance disease control efforts, improve surveillance, and response strategies. This study is one of several approaches that contribute to understanding the transmission dynamics of PPR. Strengthening biosecurity, enforcing health checks during transportation, and improving community health education are crucial for minimizing disease transmission risk.

## Figures and Tables

**Table 1:** Descriptions of selected markets for active recordings of small ruminant trade movement by volume of exchange from January to December 2023.

Survey markets	Location	Days active/week	Duration of recording	Volume of exchange (count)	
				In volume	Out volume
Bake	Borena	1 day/week	12 months	9496	11,185
Dubluk	Borena	1 day/week	12 months	9862	11,394
Elwayee	Borena	1 day/week	12 months	9772	10,277
Yabello	Borena	2 days/week	12 months	6485	3844
Deghabur	Somali	2 days/week	10 months	11,485	20,827
Gode	Somali	Whole week	10 months	30,929	35,134
Jijiga	Somali	Whole week	10 months	53,937	66,613
Kebridar	Somali	2 days/week	10 months	25,538	34,566

**Table 2:** Summary of animals' head count by destination points and means of transportation from January to December 2023.

Survey areas	Transport		Count by destination points (purpose of movement)			
			Internal market	Slaughter houses	Cross-border (export)	Total
Borena	Vehicle	Sheep	4247	7984	2602	14,833
		Goats	5325	12,097	4624	22,046
	Foot	Sheep	15,419	—	—	15,419
		Goats	19,678	—	—	19,678

Somali	Vehicle	Sheep	33,917	3612	51,289	88,818
		Goats	37,754	6534	54,623	98,911
	Foot	Sheep	36,147	—	11,690	47,837
		Goats	45,859	—	11,860	57,719

Total			198,346	30,227	136,688	365,261

**Table 3:** PPR risk status and small ruminant trade movements.

PPR risk	Number of districts	Number of markets	Number of trade links (routes)	Trade volume (animals head count)
Endemic	19	16	62	188,878
High risk	32	38	96	154,545
Relatively risk-free	12	10	13	21,838
Total	63	64	171	365,261

## Data Availability

The data that support the findings of this study are available upon request from the corresponding author.

## Supporting Information

Supporting Information It includes various data collection methods, data analysis techniques, and the construction of markets network. It also encompasses several tables and figures. Specifically, Table S1 summarizes the graph-level metrics of the small ruminant trade network, while Table S2 focuses on node-level metrics, including centrality measures of markets. Furthermore, Table S3 highlights the centrality measures of top-ranked markets in Borena and Somali livestock systems, emphasizing their connectivity contributions. The correlation between nodes' centrality measures is presented in Table S4, and Table S5 examines market centrality metrics in relation to their PPR risk status through a Kruskal—Wallis test. Visual representations include Figure S1, which illustrates the geographical links of the trade network based on activity frequency, categorized into persistent, frequent, intermediate, and occasional links. Figure S2 depicts the network degree distribution, revealing a right-skewed and heavy-tailed pattern with a few highly connected markets. The degree distributions are further analyzed in Figure S3, showcasing both unweighted and weighted degrees based on animal trading volume. The community structure is explored in Figure S4, while Figure S5 presents the largest strongly connected component of the trade network. Key actors within this network are analyzed in Figure S6, identifying markets that serve unique roles as “Pulse takers” or “Gatekeepers.” Figure S7 illustrates monthly variations in trade network structures alongside related epidemic thresholds and basic reproduction numbers (R0) throughout the year. Figures S8-S10 collectively analyze network vulnerability and spatial risk patterns: Figure S8 compares targeted node removal (by centrality) versus random removal on network connectivity through percolation analysis. Figure S9 maps 2023 PPR outbreak distributions against livestock markets, while Figure S10 specifically details PPR risk status in the Somali Region and Borena Zone alongside livestock market distributions during the same period.
