# Supplementary material for: Mapping Small Ruminant Trade Networks in Ethiopia's Somali Region and Borena Zone: Implications for the Spread of Peste des Petits Ruminants Virus
Source: Transbound Emerg Dis. 2025 Jul 13;2025:6620243. doi: 10.1155/tbed/6620243 (PMC12277055; doi:10.1155/tbed/6620243)
Supplement: Supporting Information — It includes various data collection methods, data analysis techniques, and the construction of markets network. It also encompasses several tables and figures. Specifically, Table S1 summarizes the graph-level metrics of the small ruminant trade network, while Table S2 focuses on node-level metrics, including centrality measures of markets. Furthermore, Table S3 highlights the centrality measures of top-ranked markets in Borena and Somali livestock systems, emphasizing their connectivity contributions. The correlation between nodes' centrality measures is presented in Table S4, and Table S5 examines market centrality metrics in relation to their PPR risk status through a Kruskal—Wallis test. Visual representations include Figure S1, which illustrates the geographical links of the trade network based on activity frequency, categorized into persistent, frequent, intermediate, and occasional links. Figure S2 depicts the network degree distribution, revealing a right-skewed and heavy-tailed pattern with a few highly connected markets. The degree distributions are further analyzed in Figure S3, showcasing both unweighted and weighted degrees based on animal trading volume. The community structure is explored in Figure S4, while Figure S5 presents the largest strongly connected component of the trade network. Key actors within this network are analyzed in Figure S6, identifying markets that serve unique roles as “Pulse takers” or “Gatekeepers.” Figure S7 illustrates monthly variations in trade network structures alongside related epidemic thresholds and basic reproduction numbers (R0) throughout the year. Figures S8-S10 collectively analyze network vulnerability and spatial risk patterns: Figure S8 compares targeted node removal (by centrality) versus random removal on network connectivity through percolation analysis. Figure S9 maps 2023 PPR outbreak distributions against livestock markets, while Figure S10 specifically details PPR risk status in the Somali Region and Bo [file 6620243.f1.docx]

**Supplementary Materials**

1. **Supplementary Methods**

**Data Collection Methods**

To gather relevant information on the small ruminant trade route, the quantity of movement and contact points in the network both primary and secondary data methods were applied.

### *Identification of markets for data collection*

Identification of main livestock markets for recording small ruminant movement data in the study areas was conducted by interviewing key individuals (traders and livestock office experts). Structured questionnaire-based interviews were prepared, pretested, and personnel who have more knowhow about small ruminant trade were interviewed. Each individual for the interview was selected in consultation with the regional and Zone trade and Livestock office with detailed discussions.

### *Collection of small ruminant movement data*

Data on the number of animals traded during each market day was consistently observed and recorded by individuals who were assigned for this specific purpose in all selected markets. All necessary information related to traded animals such as source and destination points, type of movement (either by truck or walking), number of animals (volume of exchange), and purpose of movement were recorded. Information about animals traded in other markets is often gathered by direct phone calls by data collectors when market days are overlapping, far distant, and difficult to attend two markets at the same time. Additionally, official animal movement records obtained from trade offices and control posts were included in data collection framework to catch the maximum possible movements and minimize possible missing in direct market recordings.

### *Retrospective epidemiological data of PPRV*

To assess the potential role of small ruminant market network structure for PPRV incursion, the study utilized retrospective data on PPR outbreaks and PPR risk status in study areas for the year2023, obtained from the Ministry of Agriculture (MoA). Since PPR is a reportable disease in Ethiopia, risk status is continuously monitored and all outbreaks of the disease must be immediately reported to MoA. Obtained data expected to provide inference of the current situation of PPR in study areas and its association with small ruminant trade network characteristics was analyzed accordingly.

1. **Supplementary Tables**

Table S1: Summary table of graph (network) level small ruminant trade network metrics

| **Network level metrics** | **Borena** | **Somali** | **Full network** |
| --- | --- | --- | --- |
| Number of nodes (Markets) | 31 | 55 | 84 |
| Number of links (Routes) | 64 | 107 | 171 |
| Density | 0.066 | 0.037 | 0.024 |
| Diameter | 3 | 5 | 5 |
| Average path length | 1.85 | 2.31 | 2.31 |
| Clustering Coefficient | 0.61 | 0.81 | 0.73 |
| Degree of assortativity | -0.55 | -0.65 | -0.52 |
| Reciprocity (%) | 6.2% | 12.3% | 10.4% |

Table S2: Summary table of node (market) level small ruminant trade network metrics (Centrality measures)

| **Centrality measures** | | **Borena** | **Somali** | **Full network** |
| --- | --- | --- | --- | --- |
| In-degree | Median | 0 | 0.5 | 0 |
|  | Max | 10 | 19 | 19 |
| Out-degree | Median | 1 | 1 | 1 |
|  | Max | 8 | 13 | 13 |
| Betweenness (average) | | 6.22 | 20.27 | 15.57 |
| Closeness(average) | | 4.35 X 10^-2^ | 2.1 X 10^-2^ | 3.51 X 10^-2^ |
| Eigenvector (average) | | 3.67 X 10^-2^ | 2.26 X 10^-1^ | 1.56 X 10^-1^ |

Table S3: Node level centrality measures of top-ranked markets and their contribution in connectivity for respective network in Borena and Somali livestock market system

| **Markets** | **Location** | **Out-degree** | **In-degree** | **Betweenness** | **Closeness** | **Eigenvector** | **% Share of connectivity** |
| --- | --- | --- | --- | --- | --- | --- | --- |
| Gode | Somali | 13 | 19 | 337.33 | 0.032 | 0.94 | 14.5% |
| Jijiga | Somali | 13 | 17 | 341.16 | 0.024 | 0.91 | 13.6% |
| Kebridar | Somali | 13 | 14 | 248.5 | 0.032 | 1 | 12.3% |
| Yabello | Borena | 7 | 10 | 60.25 | 0.14 | 0.089 | 12.9% |
| Deghabur | Somali | 7 | 8 | 157 | 0.024 | 0.67 | 6.8% |
| Dubluk | Borena | 6 | 9 | 58.08 | 0.083 | 0.066 | 11.45% |
| Bake | Borena | 7 | 7 | 49.42 | 0.091 | 0.066 | 10.7% |
| Elwayee | Borena | 8 | 6 | 25.25 | 0.1 | 0.074 | 10.7% |
| Hartshek | Somali | 4 | 4 | 8 | 0.017 | 0.039 | 3.6% |

Table S4: Correlation coefficients between nodes centrality measures

|  | **Out degree** | **In degree** | **Closeness** | **Betweenness** | **Eigenvector** |
| --- | --- | --- | --- | --- | --- |
| Out degree | 1 | 0.73 | 0.53 | 0.76 | 0.62 |
| In degree | 0.73 | 1 | 0.18 | 0.87 | 0.72 |
| Closeness | 0.53 | 0.18 | 1 | 0.13 | -0.14 |
| Betweenness | 0.76 | 0.87 | 0.13 | 1 | 0.83 |
| Eigenvector | 0.62 | 0.71 | -0.14 | 0.83 | 1 |

Table S5: Kruskal-Wallis test for markets (node) centrality metrics by their PPR risk status

| **Market (node) level centrality metrics** | **Chi squared (X^2^)** | **p-value** |
| --- | --- | --- |
| Out-degree (Outgoing links) | 7.8 | 0.020** |
| Out-weight (Outgoing volume) | 6.11 | 0.047** |
| In-degree (Incoming links) | 0.79 | 0.671 |
| In-weight (Incoming volume) | 1.23 | 0.541 |
| Betweenness | 5.15 | 0.076 |
| Closeness | 3.00 | 0.222 |
| Eigenvector | 13.66 | 0.001** |

1. **Supplementary Figures**


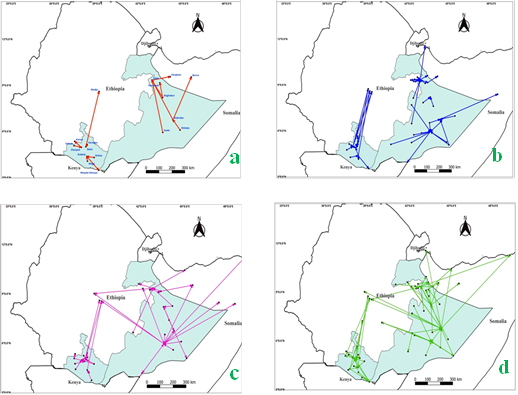


Figure S1: Geographical representation of small ruminant trade network links by frequency of activities over the year. a. persistent (active for 10 -12 months), b. frequent links (active between 6 to 9 months), c. Intermediate links (active 3 - 5 months), and d. occasional links (active only for two months or less). Black dots represent origin or destination points.


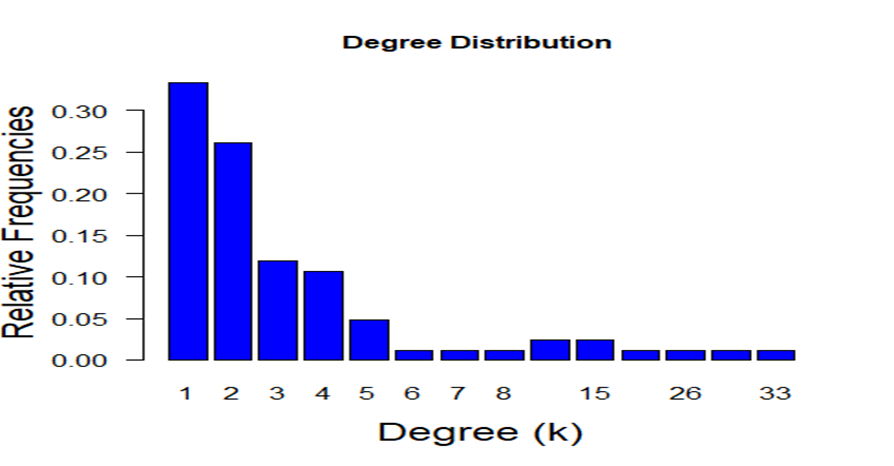


Figure S2: Network degree distribution (right skewed and heavy-tailed, with a small number of markets exhibiting relatively high degree of connectivity).


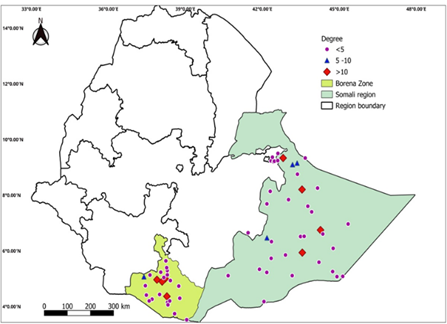

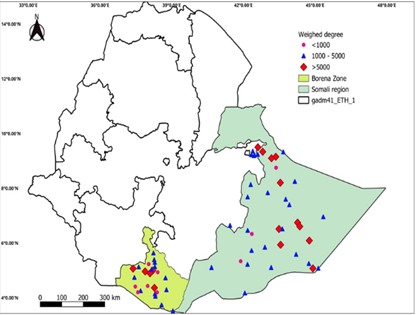


Figure S3: Maps depicting degree distributions of markets. a. un-weighed degree b. weighed degree; markets are weighed by the number of animals traded (volume of exchange)


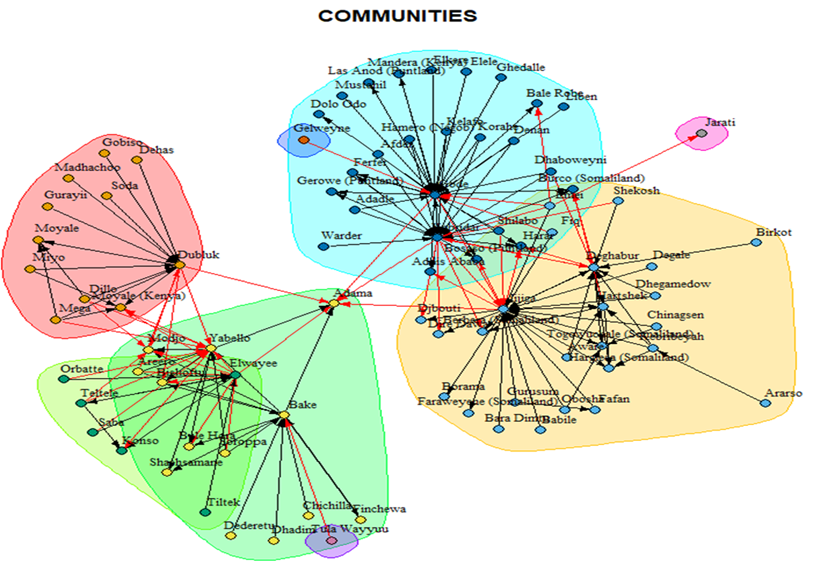


Figure S4: Community structure of small ruminant trade network in Borena and Somali region


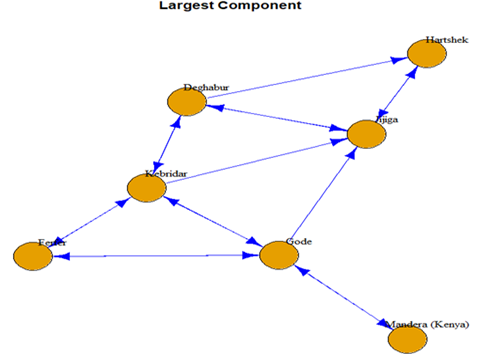


Figure S5: The largest giant strongly connected component of small ruminant trade network


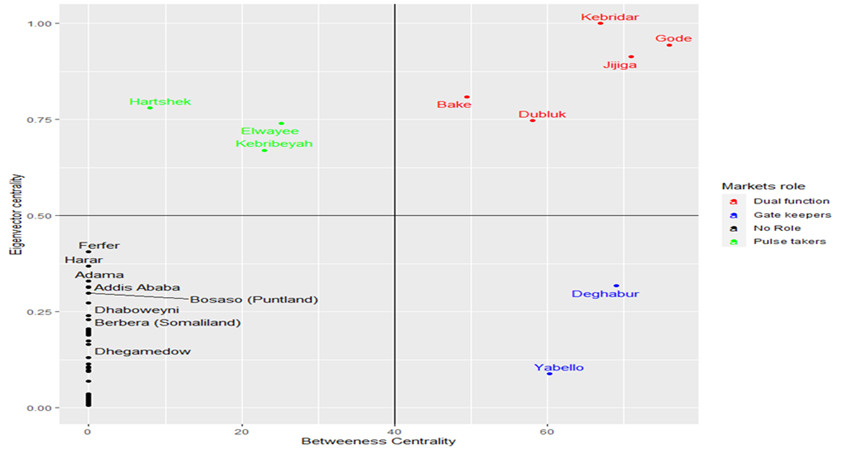


Figure S6: Key actors analysis. Markets in the top-left quadrant have unique access to central actors and are well-connected actors at the core of the network (“Pulse takers”), in the bottom-right quadrant, were being fundamental in connecting actors to the network that would otherwise be isolated from the core (“Gate keepers”), Markets in the top-right quadrant have both abilities and in the bottom left quadrant tend to have no particular role.


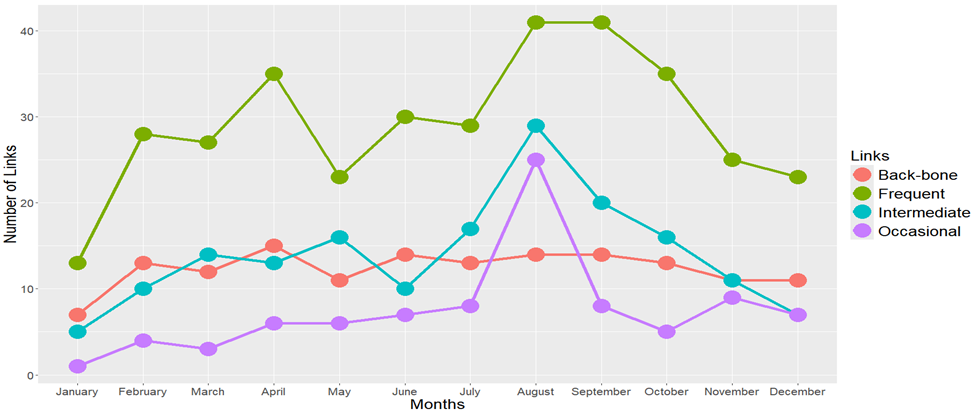

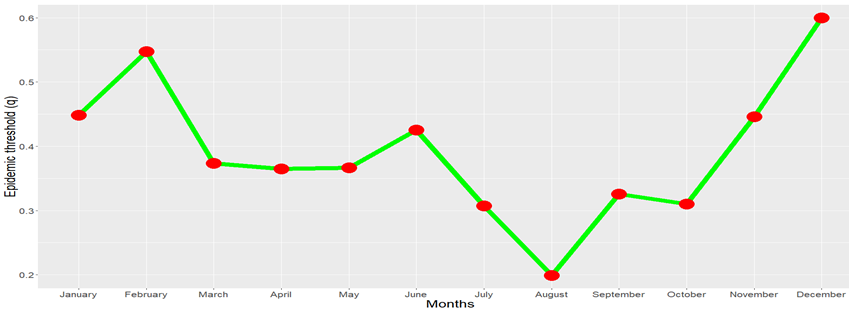

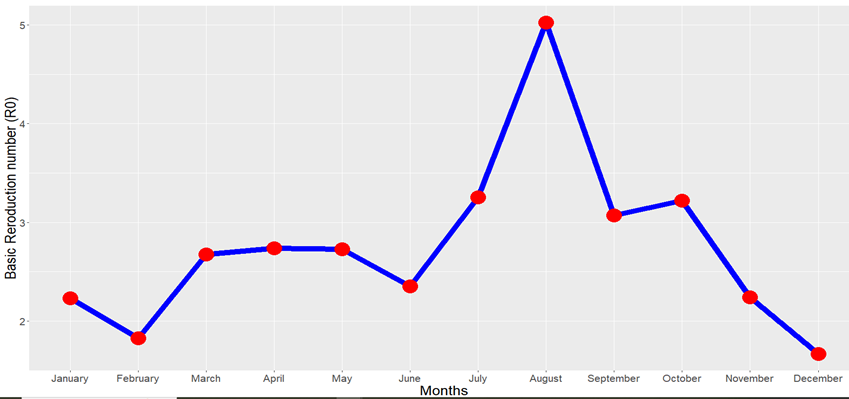


Figure S7: Monthly variations of small ruminant trade networks structures; and related epidemic threshold (*q*) and R_0_ along the year. a. Trade links, b. epidemic threshold, c. Basic reproduction number (R_0_).


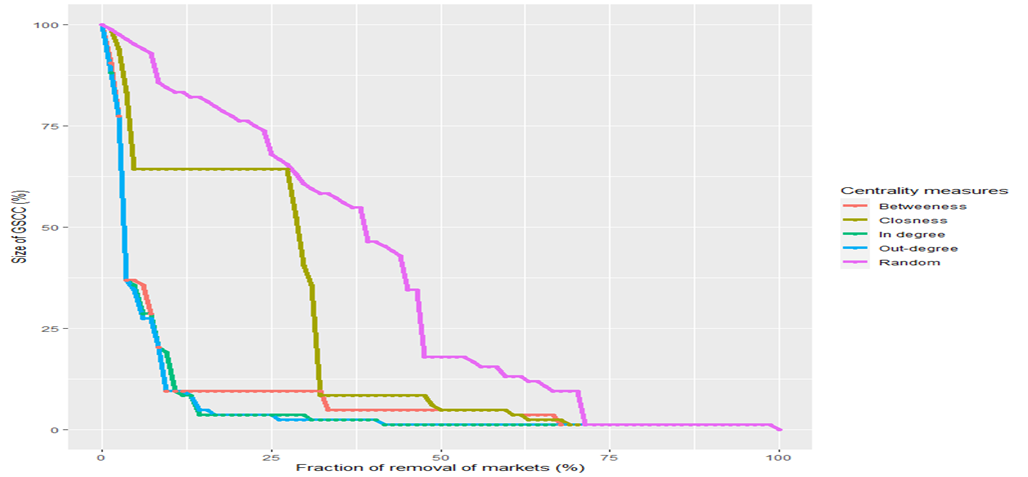


Figure S8: Plot of node centrality measures based percolation showing effect of targeted removal on connected component size compared to random removal


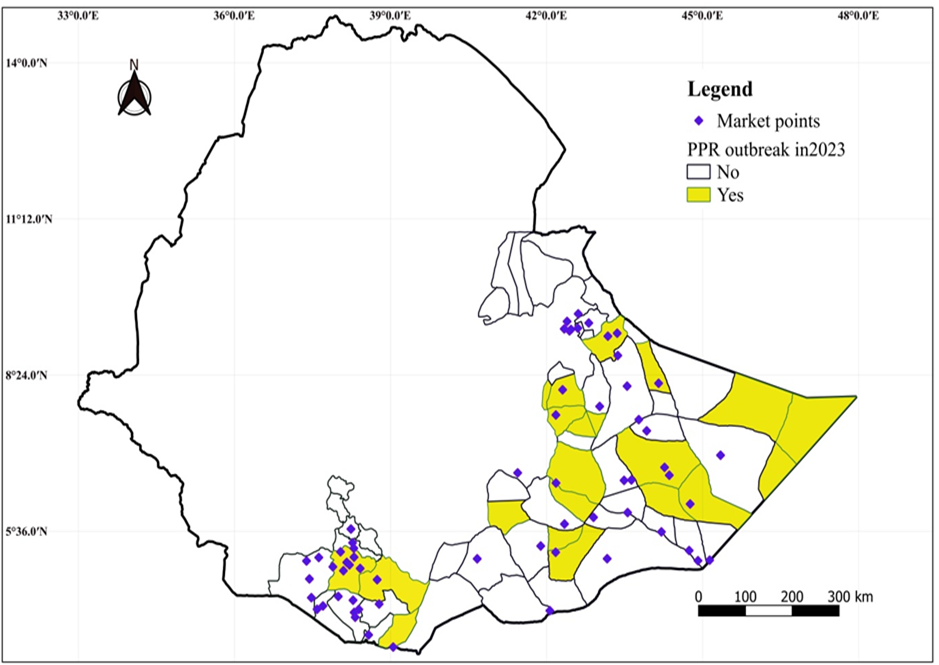


Figure S9: PPR outbreaks distributions in 2023 and livestock markets


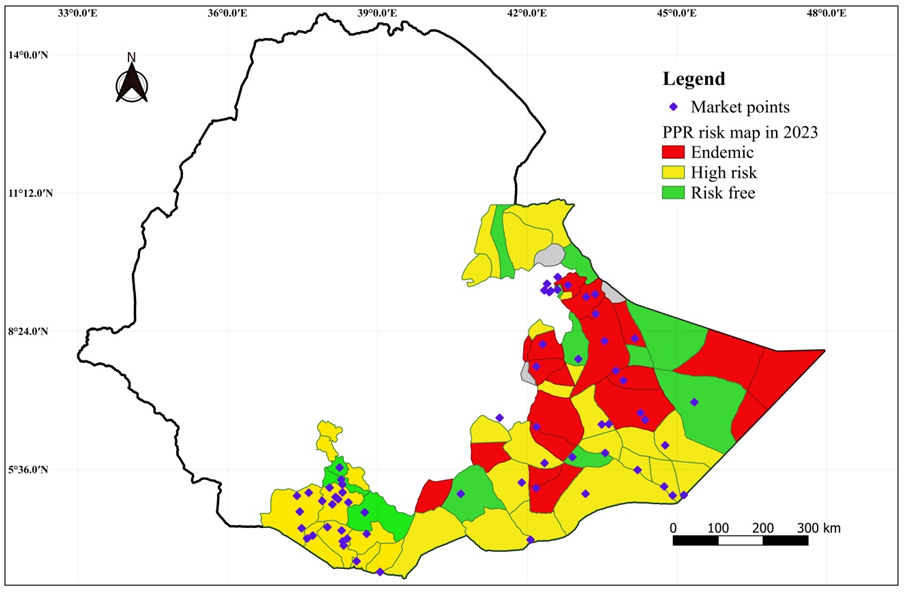


Figure S10: PPR risk status of Somali region and Borena zone in 2023 and livestock markets distribution
